# The *Chest Pain Choice *trial: a pilot randomized trial of a decision aid for patients with chest pain in the emergency department

**DOI:** 10.1186/1745-6215-11-57

**Published:** 2010-05-17

**Authors:** Meghan A Pierce, Erik P Hess, Jeffrey A Kline, Nilay D Shah, Maggie Breslin, Megan E Branda, Laurie J Pencille, Brent R Asplin, David M Nestler, Annie T Sadosty, Ian G Stiell, Henry H Ting, Victor M Montori

**Affiliations:** 1 Knowledge and Encounter Research Unit, Mayo Clinic, Rochester, Minnesota, USA; 2 Division of Emergency Medicine Research, Department of Emergency Medicine, Mayo Clinic, Rochester, Minnesota, USA; 3 Emergency Medicine Research, Department of Emergency Medicine, Carolinas Medical Center, Charlotte, NC, USA; 4 Division of Health Care Policy and Research, Department of Health Sciences Research, Mayo Clinic, Rochester, MN USA; 5 SPARC Design Studio, Center for Innovation, Mayo Clinic, Rochester, Minnesota, USA; 6 Division of Biomedical Statistics and Informatics, Department of Health Sciences Research, Mayo Clinic, Rochester, MN, USA; 7 Department of Emergency Medicine, University of Ottawa, Ottawa, Ontario, Canada; 8 Division of Cardiovascular Diseases, Department of Internal Medicine, Mayo Clinic, Rochester, MN, USA; 9 Division of Endocrinology, Diabetes, Metabolism, and Nutrition, Department of Internal Medicine, Mayo Clinic Rochester, MN, USA

## Abstract

**Background:**

Chest pain is a common presenting complaint in the emergency department (ED). Despite the frequency with which clinicians evaluate patients with chest pain, accurately determining the risk of acute coronary syndrome (ACS) and sharing risk information with patients is challenging. The aims of this study are (1) to develop a decision aid (*CHEST PAIN CHOICE*) that communicates the short-term risk of ACS and (2) to evaluate the impact of the decision aid on patient participation in decision-making and resource use.

**Methods/Design:**

This is a protocol for a parallel, 2-arm randomized trial to compare an intervention group receiving *CHEST PAIN CHOICE *to a control group receiving usual ED care. Adults presenting to the Saint Mary's Hospital ED in Rochester, MN USA with a primary complaint of chest pain who are being considered for admission for prolonged ED observation in a specialized unit and urgent cardiac stress testing will be eligible for enrollment. We will measure the effect of *CHEST PAIN CHOICE *on six outcomes: (1) patient knowledge regarding their short-term risk for ACS and the risks of radiation exposure; (2) quality of the decision making process; (3) patient and clinician acceptability and satisfaction with the decision aid; (4) the proportion of patients who decided to undergo observation unit admission and urgent cardiac stress testing; (5) economic costs and healthcare utilization; and (6) the rate of delayed or missed ACS. To capture these outcomes, we will administer patient and clinician surveys after each visit, obtain video recordings of the clinical encounters, and conduct 30-day phone follow-up.

**Discussion:**

This pilot randomized trial will develop and evaluate a decision aid for use in ED chest pain patients at low risk for ACS and provide a preliminary estimate of its effect on patient participation in decision-making and resource use.

**Trial registration:**

Clinical Trials.gov Identifier: NCT01077037

## Background

Over 6 million adults present with chest pain in US emergency departments (EDs) each year, making chest pain the second most common presenting complaint [1]. Despite the frequency with which clinicians evaluate patients with chest pain, reliably detecting patients with acute coronary syndrome (ACS) remains a diagnostic dilemma. Data from U.S. EDs suggest that 2.1% of patients with acute myocardial infarction and 2.3% with unstable angina are missed [2]. A missed diagnosis of ACS may have substantial medical and legal implications [3]. As a result, clinicians initiate formal diagnostic testing for ACS at a very low risk threshold [4]. High sensitivity is ensured at the expense of specificity, resulting in large numbers of very low risk patients being admitted to ED observation units for urgent cardiac stress testing, increased likelihood of false positive stress testing, excessive exposure to ionizing radiation [5], and significant cost to the healthcare system.

Risk communication in a busy ED setting is also a challenge. When a patient presents to the ED with chest pain and the initial evaluation for non-cardiac etiologies is unrevealing, the focus of the evaluation shifts to estimating a patient's risk for ACS. Without the assistance of decision support tools, clinicians are challenged to precisely quantify risk and often resort to terms such as "low," "rare," or "uncommon" when communicating risk to patients. One recent study reported that patients often overestimate the level of potential harm when verbal descriptors are used to communicate risk [6]. To increase patient understanding of risk and optimize knowledge transfer between physicians and patients, we will develop and evaluate a decision aid that includes a precise estimate of the short-term risk for ACS described using prose phrases, numbers, and a pictograph to account for patients' preferred mode of understanding numerical information.

To date, the authors are unaware of any published research investigating the specific role and impact of shared decision making in patients at low risk for ACS. We hypothesize that patients infrequently participate in the decision to undergo prolonged observation and urgent cardiac stress testing and that the use of a decision aid to communicate both the short-term risk of ACS and the risks of ionizing radiation exposure will increase patient involvement in the decision making process, lead to a preference-based decision, and safely decrease resource use. To test our hypothesis, we will accomplish the following specific aims: (1) develop and refine *CHEST PAIN CHOICE*, a decision aid to communicate the short-term risk for an ACS and the available management options and (2) test the effect of *CHEST PAIN CHOICE*, in a randomized clinical trial, on patient knowledge, the quality of the decision making process, patient safety, and resource use.

## Methods

### Design

To evaluate the decision aid, we will conduct a patient-level single center pilot randomized trial. The Mayo Clinic Institutional Review Board has approved all study procedures.

### Setting

Patients and emergency physicians will be recruited from the Saint Marys Hospital ED at the Mayo Clinic, a tertiary care academic ED with an annual census of 73,000 patient visits. The trial will be conducted in the flow of routine patient care. This approach will enable us to test how the decision aid performs in the real-world setting.

### Participants

#### Inclusion criteria

Eligible providers will include clinicians (attending physicians, supervised residents, nurse practitioners and physician assistants) caring for patients with chest pain. Eligible patients will include adults (>17 years of age) presenting to the ED with a primary complaint of non-traumatic chest pain who are being considered for admission to the ED observation unit for urgent cardiac stress testing.

#### Exclusion criteria

The trial will exclude patients with an initial cardiac troponin T value above the 99^th ^percentile reference limit (Roche Diagnostics [Basel, Switzerland]; 99th percentile <0.01 ng/mL; lower limit of detection 0.01 ng/mL; 10% coefficient of variation 0.035 ng/mL) [7], known coronary artery disease (at least one 50% stenosis on cardiac catheterization, electrocardiographic changes [old or not known to be old] indicative of ischemia such as ST-T changes or left bundle branch block, perfusion defects or wall motion abnormalities on exercise, pharmacologic or rest imaging studies, or previous documentation of acute myocardial infarction) [8], cocaine use within 72 hours by the clinician's initial history, and pregnancy. Patients who cannot read English or have, in their clinician's best judgment, major communication barriers such as visual or hearing impairment or dementia that would compromise their ability to give written informed consent (or use the decision aid) will be excluded.

#### Participant recruitment

We have previously developed and validated an electronic notification system that is highly sensitive and specific for identifying potentially eligible participants presenting to the ED with chest pain as a primary complaint (sensitivity 97.8%, 95% CI 96.1-98.8; specificity 98.3%, 95% CI 98.0-98.5; positive predictive value 68.8%, 95% CI 65.4-72.9; negative predictive value 99.9%, 95% CI 99.8-100.0) [9]. Shortly after arrival to the ED, a study coordinator will receive electronic notification of potentially eligible participants in real time by both pager and email. In collaboration with the treating physician, the coordinator will confirm eligibility for the trial. The coordinator will then inform patients about the trial and obtain written informed consent. Patients will be consecutively enrolled 6-7 days per week when a study coordinator is available for enrollment.

### Interventions

#### Decision aid

The *CHEST PAIN CHOICE *decision aid prototype has been developed through an iterative process involving collaboration between designers and researchers, field-testing by patients and clinicians, and revision of the decision aid in response to the feedback provided. We have employed this methodology to develop the prior decision aids *STATIN CHOICE *[10] (assists providers in discussing the benefits and side effects of statins among patients with diabetes) and *OSTEOPOROSIS CHOICE *[11] (assists with the decision to take bisphosphonates among postmenopausal women at risk for osteoporotic fracture) [12,13].

#### Selection of a risk calculator for the decision aid

**Figure 1:**
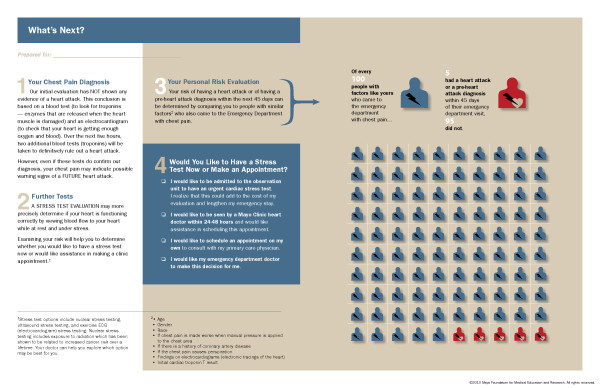
**Chest Pain Choice Decision Aid**.

We recently conducted a systematic review to identify clinical prediction tools of sufficient diagnostic accuracy and methodological quality to consider for incorporation in clinical practice [14]. Of the clinical prediction tools that have been developed for ED patients with potential ACS, only 3 have been prospectively validated and thus could be considered for use in clinical practice - the Global Registry of Acute Coronary Events (GRACE) score, the Thrombolysis in Myocardial Infarction (TIMI) risk score, and the computerized quantitative pretest probability calculator. The GRACE risk score has been validated in only one ED study, and in this study data were not available to calculate a complete risk score in 26% of patients [15]. Although the TIMI risk score has been validated in over 17,000 ED patients in 5 different countries and can provide a reliable estimate of the risk for a cardiac event within 30 days of the ED visit, it has not undergone impact analysis [16]. The pretest probability calculator developed by Kline et al., unlike the TIMI risk score, has undergone impact analysis, demonstrating its true impact on patient care [17]. The pretest probability calculator was derived from data from 14,796 patients enrolled in the multicenter internet tracking registry of ACS database and prospectively validated in 8,120 ED patients with potential ACS [18]. The pretest probability calculator has since been prospectively validated in a multicenter study of 1,114 patients from 3 different academic EDs [19] and undergone impact analysis in a practical randomized trial [17]. For these reasons, we chose to use this calculator (Figure 1) to inform our decision aid *CHEST PAIN CHOICE*.

### Randomization

We will use a dynamic allocation procedure described by Pocock and Simon [20] to randomly allocate patients via a computer-generated allocation sequence in a 1:1 concealed fashion to one of two arms: (1) a *CHEST PAIN CHOICE *arm in which the provider and patient will be provided a printout of the decision aid or (2) a usual care arm in which the provider will discuss the management options in their usual fashion without the decision aid or pretest probability calculator.

Given the limited amount of information that is available during the initial ED encounter and the known association of age and sex with cardiovascular risk, we will dynamically stratify by age and sex in the allocation process to ensure that the treatment arms are balanced in regard to these factors. To enroll patients in the trial, the study coordinator will complete the following:

• Assess patient eligibility in collaboration with the treating physician and obtain written informed consent from both parties.

• Access a secure website, input key patient characteristics, and obtain from the website interface the arm to which the patient is allocated.

After randomization, outcome assessors and data analysts will remain blind to allocation. In the event that a patient is suspected of being incorrectly enrolled (i.e., it is discovered after randomization that the patient is ineligible for the trial), the study coordinator will present the case to the principle investigator (E.P.H.) for review. The principle investigator, blinded to both allocation arm and patient outcome, will then consider post-randomization exclusion according to published recommendations [21].

The main disadvantage of randomization at the patient level is reducing the risk of contamination in the control group. Providers may be aware of the hypothesis that use of *CHEST PAIN CHOICE *will increase the quality of the decision-making process, increase patient knowledge, and decrease resource use. However, we anticipate that clinicians will revert to their usual pattern of practice without the calculator -- since access to it is password-protected -- and be reluctant to dismiss patients from the ED. We will also monitor for evidence of contamination by reviewing the video recordings of clinician-patient encounters in the control group.

#### Intervention arm

For patients randomized to the intervention arm, the study coordinator will review the contents of the decision aid in real time with the clinician immediately before the patient encounter. This approach to delivering the decision aid (with just-in-time training by the study coordinator prior to use) has been effectively used in our prior decision aid studies [22]. We have observed a trend towards superior results when the decision aid is delivered by the clinician compared to researchers delivering the tool prior to the clinical encounter [23].

#### Usual care

For patients randomized to the usual care arm, the clinicians will discuss the results of the diagnostic investigations and management options with the patient in that clinician's usual fashion. We will videotape the patient-clinician discussion for future review. No research-related interventions will be administered.

#### Outcome measures

Decision-based outcome measures take into account both the Ottawa Framework for Shared Decision Making [24] and self determination theory [25]. To assess the efficacy of the decision aid, we will measure the effect of *CHEST PAIN CHOICE *on the following outcomes: (1) patient knowledge regarding their short-term risk for ACS and the risks of radiation exposure; (2) quality of the decision making process for the patient and clinician; and (3) patient and clinician acceptability and satisfaction with the decision aid. We will also assess (4) the proportion of patients who decide to undergo observation unit admission and urgent cardiac stress testing; (5) economic costs and healthcare utilization; and (6) the rate of delayed or missed ACS (primary safety endpoint). The diagnosis of ACS will be considered delayed or missed if the patient was dismissed from the ED without observation unit admission and urgent stress testing and they meet the definition of ACS within 30 days of the ED visit. ACS will be defined as acute myocardial infarction [26], ventricular arrhythmia, cardiogenic shock, bradycardia requiring therapeutic intervention, or death attributed to a cardiac or unknown cause. Two investigators, blinded to allocation, will assess outcomes in all enrolled patients. A third investigator will resolve discordances.

A study coordinator will review the electronic medical record and conduct telephone follow-up for all enrolled patients starting at 30 days. The coordinator will verify the patient's telephone number at the time of enrollment to maximize the fidelity of follow-up. For those who are unable to be contacted by phone, we will use the resources of the Rochester Epidemiology Project [27] to determine outcomes for all Olmsted County residents and the social security death index [28] for patients who live outside of Olmsted County. We will note the success with enrollment and document the rate of 30-day follow-up.

#### Data collection

We will survey patients and clinicians after each visit and videotape the decision aid discussion between the clinician and patient in both the intervention and control groups to capture outcomes. The patient surveys (see Additional files 1 and 2 for complete list of survey questions) will include:

• Items about level of education and income level for descriptive purposes

• Questions regarding the patient's comfort level in understanding different types of numerical information (e.g., fractions, percentages, graphs)

• 7 items to assess patient knowledge, 2 of which address the risks of radiation exposure, 2 that address the available management options, 2 about the potential implications of cardiac stress test results (not informed by content on the decision aid), and one question about the patient's personalized risk for ACS within 45 days of the ED visit. These items were fashioned after similar questions, which have demonstrated construct validity and responsiveness to the intervention in previous decision aid trials [29,30].

• 5 items using a 7-point Likert-type scale to assess the patient's perceptions about the amount, clarity and helpfulness of the information, their desire to receive information about other diagnostic options in the same way this information was delivered, and whether they would recommend the way they received information from their clinician during this encounter to other patients. We have used the same items in our previous trials [11,30].

• 10 items from the Trust in Physician scale [31]

• 17 items from the Decisional Conflict Scale [32], which assesses the extent to which the decision was informed, consistent with values, free of pressure, and effective

• Additional questions that inquire about the decision made by the patient during the visit and patient satisfaction with the degree of participation in the decision making process.

The treating clinician will be asked to complete a survey after each study visit (see Additional file 3). The survey includes questions regarding which decision the patient made, the clinician's perception of who made the decision, the clinician's confidence in the patient's understanding of the information, and questions regarding the acceptability of the decision aid.

The discussion between the patient and clinician regarding the risks and benefits of further evaluation (i.e. observation unit admission with stress testing versus urgent follow-up with cardiology or follow-up with the patient's primary physician) and explanation of the *CHEST PAIN CHOICE *decision aid by the treating clinician will be video recorded to determine the degree of patient participation in the decision making process. We will use the OPTION scale [33] to evaluate the quality of the decision making on the video recordings. The scale enables an observer to quantify the extent to which clinicians involve patients in the decision making process. We have used the OPTION scale with adequate reliability in previous studies [22,34]. Video recordings will also provide information regarding the duration of the encounter (using video time stamps), fidelity of use of the tool, and difficulties encountered while using the decision aid.

We will conduct an economic evaluation of the trial using the Olmsted County Healthcare Expenditure and Utilization Database (OCHEUD). We will limit the analysis to subjects residing in Olmsted County, Minnesota since we have complete data on medical care utilization for this population. OCHEUD is a claims-based database that contains information on medical resource utilization, associated charges, and estimated economic costs for patients receiving care at either the Olmsted Medical Center or the Mayo Clinic and their associated inpatient facilities. Although the services provided represent the clinical practice patterns of Mayo Clinic and Olmsted Medical Center providers, the value of each unit of service has been adjusted to national norms by use of widely accepted valuation techniques. OCHEUD provides an estimated economic cost for each line item in the billing record and allows the aggregation of costs into categories deemed relevant to a particular study. The database serves as a major source of financial information on all patients (i.e., irrespective of payer) and includes line-item detail on date, type, frequency, and billed charge for every good or service provided. We will use data from the OCHEUD database to compare costs and utilization between the intervention and control groups. We will further compare the types of services that were used in each of the groups.

### Statistical considerations

We have sufficient funding to enroll and randomize 200 patients. While this number is insufficient to definitively assess the efficacy of the decision aid and its effect on the proportion of patients who decide to undergo observation unit admission and urgent cardiac stress testing, it is sufficient to determine the following:

• for knowledge gains, assuming the patients in the control group will, on average, answer 4 of the 7 questions correctly, we will have 90% power to detect a >25.7% increase in mean knowledge (to a mean of 5.8 questions answered correctly) in the intervention group, assuming equal variances between the two arms and alpha = 0.05;

• for the proportion of patients who decide to undergo observation unit admission and stress testing, assuming that at least 90% of patients in the control group will be admitted to the ED observation unit (null hypothesis), we will have 95% power to detect a 20% decrease in the proportion of patients who decide to be admitted to the ED observation unit for stress testing (from 90% to 70%; alternative hypothesis) in the decision aid treatment group, with a two-sided test at a 5% significance level.

The feasible sample size is also adequate to provide a plausible effect size to plan a definitive multicenter randomized trial.

We will report baseline characteristics potentially associated with study outcomes (age, gender, education level, numeracy, pretest probability for acute coronary syndrome, smoking history, proportion of patients with hypercholesterolemia, and proportion with a family history of premature coronary artery disease) separately for the intervention and control group. Each factor will be compared using either the Chi-square or Fisher's exact test for categorical variables, depending on which is appropriate for that factor. The continuous factors will be compared using the Wilcoxon rank sum test or t-test as appropriate. Any imbalance found within a risk factor will be explored as a possible factor to adjust for when the outcome measures are being analyzed. Any p-value < 0.10 will be considered a possible imbalance.

Study data will be collected and managed using REDCap electronic data capture tools hosted at the Mayo Clinic [35]. REDCap (Research Electronic Data Capture) is a secure, web-based application designed to support data capture for research studies, providing: 1) an intuitive interface for validated data entry; 2) audit trails for tracking data manipulation and export procedures; 3) automated export procedures for seamless data downloads to common statistical packages; and 4) procedures for importing data from external sources.

Continuous variables will be reported as mean (± S.D.) or medians with interquartile ranges as appropriate for the distribution of the data. Categorical data will be presented as percent frequency of occurrence. Variables will be compared using relative (relative risk) or absolute (mean differences) measures of association and their 95% confidence intervals. To test hypotheses of association, we will use Wilcoxon rank-sum tests to compare medians, and Chi-square or Fisher's exact tests to compare frequencies. All analyses will be based on two-sided tests at a significance level of 0.05. We will follow the intention to treat principle in our analysis, making every effort to avoid missing data and analyzing patients according to the arm to which they were randomized. All analyses will be conducted using SAS software (SAS institute, Inc, Carey, NC, USA) version 9.1 TS level 1M3.

## Discussion

We have described the design of a clinical trial to determine the effectiveness of a decision aid on patient participation in decision-making and resource use in ED chest pain patients at low risk for ACS. While this is a feasibility trial for a new decision aid, it will provide an estimate of the effect of the decision aid that can be used to inform the design of a larger multicenter trial.

An important reason to conduct feasibility trials of decision aids designed for use in the clinical setting is that the tools have potential to impact the nature and content of the patient-physician discussion and the duration of the clinical evaluation. Patients who present to the ED with chest pain may be in a vulnerable emotional state such that they prefer decisions be made on their behalf rather than actively participate in their medical care. The ED is also the safety net for patients of all levels of education and for those without medical insurance, and we anticipate that there may be knowledge transfer challenges that are unique to this practice setting. When clinicians with different practice patterns and level of experience use the decision aid, they may not find it appropriate to use for some patients or may not use the decision aid appropriately. Therefore, video recording the patient-clinician encounter may provide insight into these potential challenges, the effectiveness of the decision aid, and the nature of the patient-physician interaction in a busy ED environment. The results of this study may guide process changes in trial design, changes in inclusion and exclusion criteria, refinement of the decision aid itself, and data to estimate a sample size for design of a larger efficacy study.

In conclusion, there is an urgent need to develop and evaluate a shared decision making approach to patients presenting with chest pain to the ED. Chest pain is a common complaint in EDs that challenges emergency clinicians to provide an accurate, safe, and efficient diagnosis. Clinicians seem to have a very low risk tolerance and initiate diagnostic testing in patients with a very low pre-test probability of disease. Overtesting for ACS increases the likelihood of false positive stress testing, exposes patients unnecessarily to ionizing radiation, worsens ED overcrowding, and increases the cost of care. Developing and testing a decision aid that transparently communicates the short-term risk for ACS to both clinicians and patients, the potential risks of radiation exposure, and the available management options is a novel approach to solving a common clinical problem in a patient-centered fashion. Our approach differs from prior risk stratification studies of ED patients with potential ACS in that it seeks to enhance patient involvement in the decision making process rather than relying solely on diagnostic accuracy (i.e., ruling out ACS with 100% sensitivity). We hypothesize that use of *CHEST PAIN CHOICE *will increase patient knowledge and involvement in the decision making process, decrease physician and patient anxiety, and safely decrease resource use in ED patients with chest pain. We will also continue to develop the expertise of our group in the conduct of decision aid trials in complex real-world settings.

## List of abbreviations

ED: emergency department; ACS: acute coronary syndrome; GRACE: Global Registry of Acute Coronary Events; TIMI: Thrombolysis in myocardial infarction; OCHEUD: Olmsted County Healthcare Expenditure and Utilization Database; REDCap: Research Electronic Data Capture;

## Competing interests

The authors declare that they have no competing interests.

## Authors' contributions

MAP contributed to the design of the study, designed study procedures, and wrote the first draft of the manuscript. EPH made substantial contributions to the conception and design of the study and the study protocol, applied for funding, and made critical revisions to the manuscript. JAK made substantial contributions to the conception and design of the study, developed the pretest probability calculator to inform the decision aid, and made critical revisions to the manuscript. NDS contributed to the conception and design of the study, wrote the economic analysis portion of the manuscript, and provided critical revisions to other sections of the manuscript. MB contributed to the conception and design of the study, designed the decision aid, and provided critical revisions to the manuscript. MEB contributed to the statistical analysis of the study, wrote the statistical section of the manuscript, and provided critical revisions to other sections of the manuscript. LJP contributed to the design of the study, designed study procedures, and provided critical revisions to the manuscript. BRA, DMN, ATS, and IGS contributed to the conception and design of the study and made critical revisions to the manuscript. HHT, a cardiologist, provided subspecialty expertise to the conception and design of the study and made critical revisions to the manuscript. VMM made substantial contributions to the conception and design of the study and the study protocol and made critical revisions to the manuscript. All authors approved the final version of this manuscript.

## Supplementary Material

Additional file 1 General patient survey. Click here for file

Additional file 2 Wiser choices patient survey (numeracy survey). Click here for file

Additional file 3 Provider Survey. Click here for file
