# Supplementary material for: The Chest Pain Choice trial: a pilot randomized trial of a decision aid for patients with chest pain in the emergency department
Source: Trials. 2010 May 17;11:57. doi: 10.1186/1745-6215-11-57 (PMC2881067; doi:10.1186/1745-6215-11-57)
Supplement: Additional file 1 — General patient survey. [file 1745-6215-11-57-S1.DOC]

*Chest Pain Choice*

*Patient Survey*

| **INSTRUCTIONS: PLEASE CHECK THE APPROPRIATE BOX OR FILL IN THE BLANK AS INDICATED.** |
| --- |

| **The following questions are about you, your chest pain symptoms, and your knowledge of your risk for a heart attack or a pre-heart attack diagnosis within the next 45 days. Please mark the best answer to each of these questions by putting an X over the number you select.** |
| --- |

| **A1.** | **How would you describe the *amount* of information about your risk for a heart attack or pre-heart attack diagnosis given during this visit?** |
| --- | --- |

| 1 ❑ | 2 ❑ | 3 ❑ | 4 ❑ | 5 ❑ | 6 ❑ | 7 ❑ |
| --- | --- | --- | --- | --- | --- | --- |
| Too little information |  |  | Just the right amount of information |  |  | Too much information |

| **A2.** | 1. **How would you describe the *clarity* of information about the risk of a heart attack or pre-heart attack diagnosis given during this visit?** |
| --- | --- |

| 1 ❑ | 2 ❑ | 3 ❑ | 4 ❑ | 5 ❑ | 6 ❑ | 7 ❑ |
| --- | --- | --- | --- | --- | --- | --- |
| Not clear at all |  |  | Somewhat clear |  |  | Extremely clear |

| **A3.** | **How *helpful* was the information about a heart attack or pre-heart attack diagnosis that you were given during this visit?** |
| --- | --- |

| 1 ❑ | 2 ❑ | 3 ❑ | 4 ❑ | 5 ❑ | 6 ❑ | 7 ❑ |
| --- | --- | --- | --- | --- | --- | --- |
| Not helpful at all |  |  | Somewhat helpful |  |  | Extremely helpful |

| **A4.** | 1. **Would you want to get information about other diagnostic options in the same way that you got information about your risk for a heart attack or pre-heart attack diagnosis during this visit?** |
| --- | --- |

| 1 ❑ | 2 ❑ | 3 ❑ | 4 ❑ | 5 ❑ | 6 ❑ | 7 ❑ |
| --- | --- | --- | --- | --- | --- | --- |
| Yes, for sure |  |  | Not sure |  |  | No, not at all |

| **A5.** | **Would you recommend the way that you and your provider shared information about your risk for a heart attack or pre-heart attack diagnosis to other patients?** |
| --- | --- |

| 1 ❑ | 2 ❑ | 3 ❑ | 4 ❑ | 5 ❑ | 6 ❑ | 7 ❑ |
| --- | --- | --- | --- | --- | --- | --- |
| Yes, I strongly recommend it |  |  | Not sure whether to recommend it or not |  |  | No, I strongly recommend against it |

| **B. Thinking about your visit with your provider today, please look at the following comments made by some people when deciding about what workup to undergo to rule out a heart attack or pre-heart attack diagnosis.** |
| --- |

|  |  | Strongly agree | Agree | Neither agree nor disagree | | Disagree | Strongly disagree |
| --- | --- | --- | --- | --- | --- | --- | --- |
|  |  |  |  | |  |  |  |

| **B1.** | **My provider identified the choice of being admitted to the observation unit and having a cardiac stress test as a decision that I could participate in** | 1 ❑ | 2 ❑ | 3 ❑ | 4 ❑ | 5 ❑ |
| --- | --- | --- | --- | --- | --- | --- |

| **B2.** | **My provider explained to me the pros and cons of observation unit admission and cardiac stress testing** | 1 ❑ | 2 ❑ | 3 ❑ | 4 ❑ | 5 ❑ |
| --- | --- | --- | --- | --- | --- | --- |

| **B3.** | **My provider asked about my expectations (or ideas) about having a cardiac stress test to rule out a pre-heart attack diagnosis** | 1 ❑ | 2 ❑ | 3 ❑ | 4 ❑ | 5 ❑ |
| --- | --- | --- | --- | --- | --- | --- |

| **B4.** | **My provider asked about my concerns (or fears) about having a cardiac stress test to rule out a pre-heart attack diagnosis** | 1 ❑ | 2 ❑ | 3 ❑ | 4 ❑ | 5 ❑ |
| --- | --- | --- | --- | --- | --- | --- |

| **B5.** | **My provider asked me whether I understood the information he/she gave me about my risk for a heart attack or pre-heart attack diagnosis** | 1 ❑ | 2 ❑ | 3 ❑ | 4 ❑ | 5 ❑ |
| --- | --- | --- | --- | --- | --- | --- |

|  |  | Strongly agree | Agree | Neither agree nor disagree | Disagree | Strongly disagree |
| --- | --- | --- | --- | --- | --- | --- |
|  |  |  |  |  |  |  |
| **B6.** | **My provider gave me the opportunity to make the decision about having a stress test now or following up with a heart doctor** | 1 ❑ | 2 ❑ | 3 ❑ | 4 ❑ | 5 ❑ |

| **B7.** | **My provider gave me the option to reconsider the decision about having an urgent cardiac stress test in the future** | 1 ❑ | 2 ❑ | 3 ❑ | 4 ❑ | 5 ❑ |
| --- | --- | --- | --- | --- | --- | --- |

| **C. Please answer the following questions to the best of your ability. *This is not a test; what is important is that your answers reflect what you think is true.*** |
| --- |

|  |  | True  Yes | False  No | Don’t know |
| --- | --- | --- | --- | --- |
|  |  |  |  |  |

| **C1.** | **If the result of the cardiac stress test we discussed is negative, there is an increased certainty that my chest pain is *not* due to my heart..** | 1 ❑ | 2 ❑ | 3 ❑ |
| --- | --- | --- | --- | --- |

| **C2.** | **If the result of the cardiac stress test we discussed is positive, this may be a false positive and lead to additional testing that is unnecessary.** | 1 ❑ | 2 ❑ | 3 ❑ |
| --- | --- | --- | --- | --- |

| **C3.** | **None of the cardiac stress tests we discussed expose me to radiation** | 1 ❑ | 2 ❑ | 3 ❑ |
| --- | --- | --- | --- | --- |

| **C4.** | **I understand that I can follow-up with a heart specialist (cardiologist) within 24-48 hours for additional evaluation.** | 1 ❑ | 2 ❑ | 3 ❑ |
| --- | --- | --- | --- | --- |

| **C5.** | **I understand that I can follow-up with my own doctor at the next available visit for additional evaluation if that is my preference** | 1 ❑ | 2 ❑ | 3 ❑ |
| --- | --- | --- | --- | --- |

| **C6.** | **Radiation exposure may increase my lifetime risk for cancer.** | 1 ❑ | 2 ❑ | 3 ❑ |
| --- | --- | --- | --- | --- |

| **C7.** | **Of 100 people like you, how many do you think will develop a heart attack or pre-heart attack diagnosis within the next 45 days?**  **______** |
| --- | --- |

|  | **Questions C1 through C7:** © Copyright Mayo Foundation. |
| --- | --- |

| **D. How much do you trust the provider who discussed diagnostic options for ruling out your risk for a heart attack or pre-heart attack diagnosis to :** |
| --- |

|  |  | Completely | Mostly | | Somewhat | A little | | Not at all |
| --- | --- | --- | --- | --- | --- | --- | --- | --- |
|  |  |  | |  |  |  |  | |

| **D1.** | **Always tell you the truth** | 1 ❑ | 2 ❑ | 3 ❑ | 4 ❑ | 5 ❑ |
| --- | --- | --- | --- | --- | --- | --- |

| **D2.** | **Provide you with accurate, up-to-date, medical information** | 1 ❑ | 2 ❑ | 3 ❑ | 4 ❑ | 5 ❑ |
| --- | --- | --- | --- | --- | --- | --- |

| **D3.** | **Make it easy for you to bring up a prior discussion about your condition and discuss it again** | 1 ❑ | 2 ❑ | 3 ❑ | 4 ❑ | 5 ❑ |
| --- | --- | --- | --- | --- | --- | --- |

| **D4.** | **Make excellent medical judgments on your behalf** | 1 ❑ | 2 ❑ | 3 ❑ | 4 ❑ | 5 ❑ |
| --- | --- | --- | --- | --- | --- | --- |

| **D5.** | **Do everything medically that should be done in order to ensure the best possible result** | 1 ❑ | 2 ❑ | 3 ❑ | 4 ❑ | 5 ❑ |
| --- | --- | --- | --- | --- | --- | --- |

|  |  | Completely | Mostly | Somewhat | A little | Not at all |
| --- | --- | --- | --- | --- | --- | --- |
|  |  |  |  |  |  |  |
| **D6.** | **Tell you when you could benefit from seeing a specialist** | 1 ❑ | 2 ❑ | 3 ❑ | 4 ❑ | 5 ❑ |

| **D7.** | **Tell you if a mistake was made about your treatment** | 1 ❑ | 2 ❑ | 3 ❑ | 4 ❑ | 5 ❑ |
| --- | --- | --- | --- | --- | --- | --- |

| **D8.** | **Put your medical needs above all other considerations, including cost** | 1 ❑ | 2 ❑ | 3 ❑ | 4 ❑ | 5 ❑ |
| --- | --- | --- | --- | --- | --- | --- |

| **D9.** | **Listen well so he/she understands your needs and concerns** | 1 ❑ | 2 ❑ | 3 ❑ | 4 ❑ | 5 ❑ |
| --- | --- | --- | --- | --- | --- | --- |

| **D10.** | **Never pretend to know things when he/she is not sure** | 1 ❑ | 2 ❑ | 3 ❑ | 4 ❑ | 5 ❑ |
| --- | --- | --- | --- | --- | --- | --- |

| **Questions E1 through E10:** Used with the permission from Thom DH, Kravitz RL, Bell RA, Krupat E, Azari R. |
| --- |

| **E1.** | **Which option did you decide today?** |
| --- | --- |

| 1 ❑ | **To be admitted to the observation unit and have an urgent cardiac stress test.** |
| --- | --- |

| 2 ❑ | **To follow-up with a Mayo Clinic heart doctor within 24-72 hours.** |
| --- | --- |

| 3❑ | **To follow-up with my own primary care physician at the next available appointment** |
| --- | --- |

| 4 ❑ | **To have the emergency doctor make the decision for me** |
| --- | --- |

| **F. Now, thinking about the choice you made, please look at the following comments made by some people when deciding about whether to be admitted to the observation unit and have an urgent cardiac stress test. Please show how strongly you agree or disagree with these statements by checking the box that indicates your level of agreement.** |
| --- |

|  |  | Strongly agree | Agree | Neither agree nor disagree | Disagree | Strongly disagree |
| --- | --- | --- | --- | --- | --- | --- |
|  |  |  |  |  |  |  |

| **F1.** | **I expect to stick with my decision** | 1 ❑ | 2 ❑ | 3 ❑ | 4 ❑ | 5 ❑ |
| --- | --- | --- | --- | --- | --- | --- |

| **F2.** | **My provider and I agreed about which treatment (or no treatment) was best for me** | 1 ❑ | 2 ❑ | 3 ❑ | 4 ❑ | 5 ❑ |
| --- | --- | --- | --- | --- | --- | --- |

| **F3.** | **I know which options are available to me** | 1 ❑ | 2 ❑ | 3 ❑ | 4 ❑ | 5 ❑ |
| --- | --- | --- | --- | --- | --- | --- |

| **F4.** | **I know the benefits of each option** | 1 ❑ | 2 ❑ | 3 ❑ | 4 ❑ | 5 ❑ |
| --- | --- | --- | --- | --- | --- | --- |
|  |  |  |  |  |  |  |
| **F5.** | **I know the risks of each option** | 1 ❑ | 2 ❑ | 3 ❑ | 4 ❑ | 5 ❑ |

| **F6.** | **I am clear about which benefits matter most to me** | 1 ❑ | 2 ❑ | 3 ❑ | 4 ❑ | 5 ❑ |
| --- | --- | --- | --- | --- | --- | --- |

| **F7.** | **I am clear about which risks matter most to me** | 1 ❑ | 2 ❑ | 3 ❑ | 4 ❑ | 5 ❑ |
| --- | --- | --- | --- | --- | --- | --- |

| **F8.** | **I am clear about which is more important to me (the benefits or the risks and side effects)** | 1 ❑ | 2 ❑ | 3 ❑ | 4 ❑ | 5 ❑ |
| --- | --- | --- | --- | --- | --- | --- |

| **F9.** | **I have enough support from others to make a choice** | 1 ❑ | 2 ❑ | 3 ❑ | 4 ❑ | 5 ❑ |
| --- | --- | --- | --- | --- | --- | --- |

|  |  | Strongly agree | Agree | Neither agree nor disagree | Disagree | Strongly disagree |
| --- | --- | --- | --- | --- | --- | --- |
|  |  |  |  |  |  |  |

| **F10.** | **I am choosing without pressure from others** | 1 ❑ | 2 ❑ | 3 ❑ | 4 ❑ | 5 ❑ |
| --- | --- | --- | --- | --- | --- | --- |

| **F11.** | **I have enough advice to make a choice** | 1 ❑ | 2 ❑ | 3 ❑ | 4 ❑ | 5 ❑ |
| --- | --- | --- | --- | --- | --- | --- |

| **F12.** | **I am clear about the best choice for me** | 1 ❑ | 2 ❑ | 3 ❑ | 4 ❑ | 5 ❑ |
| --- | --- | --- | --- | --- | --- | --- |

| **F13.** | **I feel sure about what to choose** | 1 ❑ | 2 ❑ | 3 ❑ | 4 ❑ | 5 ❑ |
| --- | --- | --- | --- | --- | --- | --- |

| **F14.** | **This decision is easy for me to make** | 1 ❑ | 2 ❑ | 3 ❑ | 4 ❑ | 5 ❑ |
| --- | --- | --- | --- | --- | --- | --- |

| **F15.** | **I feel I have made an informed choice** | 1 ❑ | 2 ❑ | 3 ❑ | 4 ❑ | 5 ❑ |
| --- | --- | --- | --- | --- | --- | --- |

| **F16.** | **The decision shows what is important to me** | 1 ❑ | 2 ❑ | 3 ❑ | 4 ❑ | 5 ❑ |
| --- | --- | --- | --- | --- | --- | --- |

| **F17.** | **I am satisfied with my decision** | 1 ❑ | 2 ❑ | 3 ❑ | 4 ❑ | 5 ❑ |
| --- | --- | --- | --- | --- | --- | --- |

| **Questions F1 through F17:** ©Annette O’Connor, revised 2005, adaptation to assess risk for an acute coronary syndrome |
| --- |

| **G1.** | **Thinking about the discussion you had today about whether to admitted to the observation unit and have a stress test, how was the decision made?** |
| --- | --- |

| 1 ❑ | **You made the decision** |
| --- | --- |

| 2 ❑ | **It was a shared decision between the doctor and me** |
| --- | --- |

| 3❑ | **The doctor made the decision for me** |
| --- | --- |

| **H1.** | **My decision to about whether to be admitted to the observation unit and have an urgent cardiac stress test or not would be different if *cost* was *not* an issue. In other words, *if the workup was free, or very inexpensive, would your decision be different?*** |
| --- | --- |

| 1 ❑ | **Yes** |
| --- | --- |

| 2 ❑ | **No** |
| --- | --- |

| **I1.** | **What is the highest level of schooling you have completed?** | | |
| --- | --- | --- | --- |
| 1 ❑ | | **8^th^ grade or less** |  |

| 2 ❑ | **Some high school** |
| --- | --- |

| 3 ❑ | **High school graduate or GED** |
| --- | --- |

| 4 ❑ | **Some high college or vocational school** |
| --- | --- |

| 5 ❑ | **College graduate (4 year college)** |
| --- | --- |

| 6 ❑ | **Graduate degree** |
| --- | --- |

| **I1.** | **Which of the following categories best describes your household income last year?** |
| --- | --- |

| 1 ❑ | **Less than $20,000** |
| --- | --- |

| 2 ❑ | **$20,000 to $30,000** |
| --- | --- |

| 3 ❑ | **$30,000 to $40,000** |
| --- | --- |

| 4 ❑ | **$40,000 to $60,000** |
| --- | --- |

| 5 ❑ | **$60,000 to $80,000** |
| --- | --- |

| 6 ❑ | **$80,000 to $100,000** |
| --- | --- |

| 7 ❑ | **$100,000 or more** |
| --- | --- |

**Comment**

|  |
| --- |
|  |
|  |
|  |
|  |
