# Supplementary material for: The Chest Pain Choice trial: a pilot randomized trial of a decision aid for patients with chest pain in the emergency department
Source: Trials. 2010 May 17;11:57. doi: 10.1186/1745-6215-11-57 (PMC2881067; doi:10.1186/1745-6215-11-57)
Supplement: Additional file 2 — Wiser choices patient survey (numeracy survey). [file 1745-6215-11-57-S2.PDF]

MAYO CLINIC

Wiser Choices 1
Patient Survey

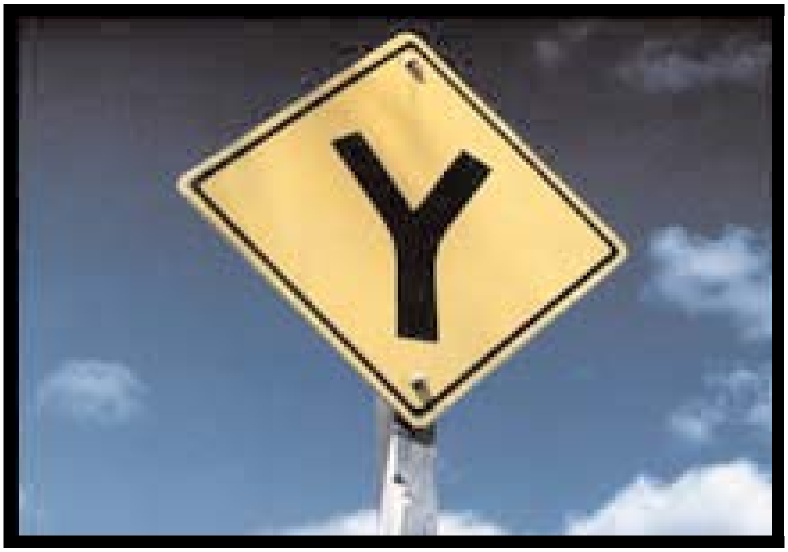

Clinic Number:..

Date:..

For each of the following questions, please check the box that best reflects **how good you are at doing the following things**:

1. How good are you at working with fractions?

1	2	3	4	5	6
**Not at all good**					**Extremely good**

2. How good are you at working with percentages?

1	2	3	4	5	6
**Not at all good**					**Extremely good**

3. How good are you at calculating a 15% tip?

1	2	3	4	5	6
**Not at all good**					**Extremely good**

4. How good are you at figuring out how much a shirt will cost if it is 25% off?

1	2	3	4	5	6
**Not at all good**					**Extremely good**

For each of the following questions, please check the box that **best reflects your answer:**

5. When reading the newspaper, how **helpful** do you find tables and graphs that are parts of a story?

1	2	3	4	5	6
**Not at all helpful**					**Extremely helpful**

6. When people tell you the chance of something happening, do you prefer that they use **words** ("it rarely happens") or **numbers** ("there's a 1% chance")?

1	2	3	4	5	6
**Always Prefer Words**					**Always Prefer Numbers**

7. When you hear a weather forecast, do you prefer predictions using **percentages** (e.g., “there will be a 20% chance of rain today”) or predictions using only **words** (e.g., “there is a small chance of rain today”)?

1	2	3	4	5	6
**Always Prefer Percentages**					**Always Prefer Words**

8. How **often** do you find numerical information to be useful?

1	2	3	4	5	6
**Never**					**Very Often**

Questions 1-8: © B.J. Zikmund-Fisher, D.M. Smith, P.A. Ubel, and A. Fagerlin. 2007

MAYO CLINIC

200 First Street SW
Rochester, Minnesota 55905
www.mayoclinic.org

© Mayo Foundation for Medical Education and Research (MFMER). All rights reserved.
MAYO, MAYO CLINIC and the triple-shield Mayo logo are trademarks and service marks of MFMER.
