# Supplementary material for: The Chest Pain Choice trial: a pilot randomized trial of a decision aid for patients with chest pain in the emergency department
Source: Trials. 2010 May 17;11:57. doi: 10.1186/1745-6215-11-57 (PMC2881067; doi:10.1186/1745-6215-11-57)

Chest Pain Choice:

Provider Survey

Knowledge and Encounter Research Unit

**Physician Name:**

**Patient Name and MRN #:**

**Date:**

| **A1.** | **Which decision did your patient make today?** |
| --- | --- |

| 1 ❑ | **To be admitted to the observation unit and have an urgent cardiac stress test.** |
| --- | --- |

| 2 ❑ | **To follow-up with a Mayo Clinic cardiologist within 24-72 hours** |
| --- | --- |

| 3❑ | **To follow-up with their own primary care physician at the next available appointment** |
| --- | --- |

| **A2.** | **Thinking about the discussion you had today about whether to be admitted to the observation unit and have a stress test, how was the decision made?** |
| --- | --- |

| 1 ❑ | **The patient made the decision** |
| --- | --- |

| 2 ❑ | **It was a shared decision between me and the patient** |
| --- | --- |

| 3❑ | **I made the decision (patient deferred to you)** |
| --- | --- |

|  |  | Strongly agree | Agree | Neither agree nor disagree | Disagree | Strongly disagree |
| --- | --- | --- | --- | --- | --- | --- |
|  |  |  |  |  |  |  |

| **3.** | **I feel the patient has made an informed choice** | 1 ❑ | 2 ❑ | 3 ❑ | 4 ❑ | 5 ❑ |
| --- | --- | --- | --- | --- | --- | --- |

| **4.** | **The patient’s decision shows what is important to him/her** | 1 ❑ | 2 ❑ | 3 ❑ | 4 ❑ | 5 ❑ |
| --- | --- | --- | --- | --- | --- | --- |

| **5.** | **I expect the patient to stick with his/her decision** | 1 ❑ | 2 ❑ | 3 ❑ | 4 ❑ | 5 ❑ |
| --- | --- | --- | --- | --- | --- | --- |

| **6.** | **I think the patient is satisfied with his/her decision** | 1 ❑ | 2 ❑ | 3 ❑ | 4 ❑ | 5 ❑ |
| --- | --- | --- | --- | --- | --- | --- |

| **Questions 3 through 6:** © Annette O. Connor, revised 2005, adaptation to chest pain choice survey |
| --- |
|  |

| **B1.** | **You gave information about acute coronary syndrome, the patient’s risk for ACS, and their diagnostic options during this visit. How *helpful* do you think this information was to the patient?** |
| --- | --- |

| 1 ❑ | 2 ❑ | 3 ❑ | 4 ❑ | 5 ❑ | 6 ❑ | 7 ❑ |
| --- | --- | --- | --- | --- | --- | --- |
| Not helpful at all |  |  | Somewhat helpful |  |  | Extremely helpful |

| **B2.** | **Would you *recommend* to other providers the way that you presented information about ACS, the patient’s risk for ACS, and their diagnostic options during this visit?** |
| --- | --- |

| 1 ❑ | 2 ❑ | 3 ❑ | 4 ❑ | 5 ❑ | 6 ❑ | 7 ❑ |
| --- | --- | --- | --- | --- | --- | --- |
| No, I would strongly recommend against it |  |  | Not sure whether to recommend it or not |  |  | Yes, I would strongly recommend it |

| **B3.** | 1. **Would you want to present information about other diagnostic choices in the same way that you presented information about acute coronary syndrome during this visit?** |
| --- | --- |

| 1 ❑ | 2 ❑ | 3 ❑ | 4 ❑ | 5 ❑ | 6 ❑ | 7 ❑ |
| --- | --- | --- | --- | --- | --- | --- |
| No, not at all |  |  | Not sure |  |  | Yes, for sure |


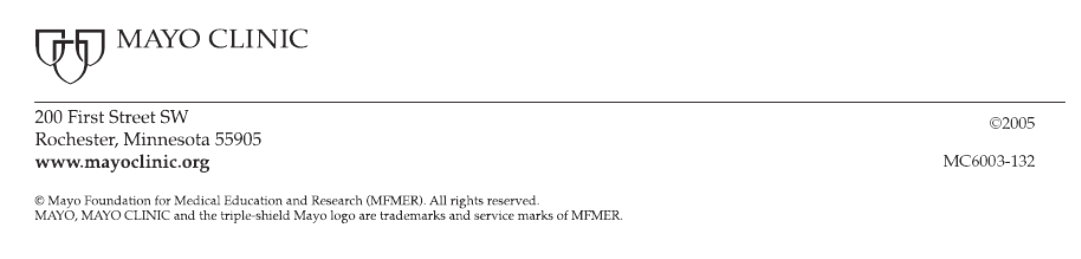

Supplement: Additional file 3 — Provider Survey. [file 1745-6215-11-57-S3.DOC]
